# Failure to prevent classical scrapie after repeated decontamination of a barn

**DOI:** 10.1186/s13104-025-07188-1

**Published:** 2025-03-25

**Authors:** Timm Konold, John Spiropoulos, Peter Bellerby, Hugh A Simmons

**Affiliations:** 1 Animal and Plant Health Agency Weybridge, Department of Pathology and Animal Sciences, Addlestone, UK; 2 Animal and Plant Health Agency Weybridge, National Science Centre for Animal Health, Addlestone, UK

**Keywords:** Scrapie, Sheep, Prion, Transmissible spongiform encephalopathy, Decontamination, Sodium hypochlorite, Bioassay

## Abstract

**Objective:**

Prions, the causative agent of scrapie in sheep, are extremely resistant to disinfection and can remain biologically active for years, which makes it challenging to prevent re-infection of susceptible animals on farms after a scrapie outbreak. The present study investigated the effectiveness of disinfection of a barn that previously housed scrapie-affected sheep as part of the husbandry of scrapie infected sheep on the farm. The barn was decontaminated with sodium hypochlorite for four times the recommended exposure time. Two cohorts, consisting of 25 and 21 sheep, with susceptible prion protein genotypes (VRQ/VRQ), born 2 years apart, were housed in the barn and infection monitored by examination of rectal biopsies.

**Results:**

One sheep from the first cohort and four from the second were found to be infected from 775 (first cohort) and 550 days (second cohort) post exposure. It is concluded that decontamination with sodium hypochlorite at the recommended concentration and longer exposure time did not prevent re-infection of susceptible sheep. Disinfection of contaminated premises to eradicate scrapie continues to be a challenge.

**Supplementary Information:**

The online version contains supplementary material available at 10.1186/s13104-025-07188-1.

## Introduction

Classical scrapie is a transmissible spongiform encephalopathy and neurogenerative disease in sheep and goats caused by the accumulation of proteinase-resistant prion protein in the brain. Prions are known to be extremely difficult to inactivate and can persist in the environment for many years without losing their ability to infect susceptible species. Current guidelines for the disinfection of premises that were occupied by sheep with classical scrapie advise the use of sodium hypochlorite at a concentration of at least 20,000 parts per million active chlorine with a contact time of at least 1 h [1]. A study has shown that this protocol is inadequate to prevent re-infection if new sheep are introduced [2], but laboratory studies using an ultrasensitive prion detection test, serial protein misfolding cyclic amplification (sPMCA), suggested that the biological activity of prions is greatly reduced on concrete surfaces if the disinfection process with sodium hypochlorite for one hour is repeated three times [3]. However, this protocol when applied to a farm building still could not prevent re-infection of sheep, even though the building was not considered to be contaminated based on sPMCA on environmental swabs. In fact, all 24 lambs that were housed in the barn, excluding one that died at 122 days post exposure (dpe), had scrapie confirmed by postmortem tests [4]. The most likely explanation was that dust from the surrounding environment led to re-contamination since prions were detected in dust samples collected over time, which was independent of occupancy with sheep because prions were even detected in an adjacent barn that did not house sheep. This could imply that dust from the outside containing the scrapie agent is likely to have entered the barn and caused infection of sheep.

The objective of the follow-up study described here was to determine whether subsequent disinfection using the same protocol, combined with the weathering process whilst the surrounding area outside the barn was not inhabited by sheep for more than a year, would eventually prevent re-infection of sheep.

## Methods

All animal experiments were carried out under project licences (P97FF6D49, P1B421A0B8) approved by the UK Home Office. This is only granted after ethical approval has been given by the Animal Welfare and Ethical Review Body of the Animal and Plant Health Agency (APHA).

**Fig. 1:**
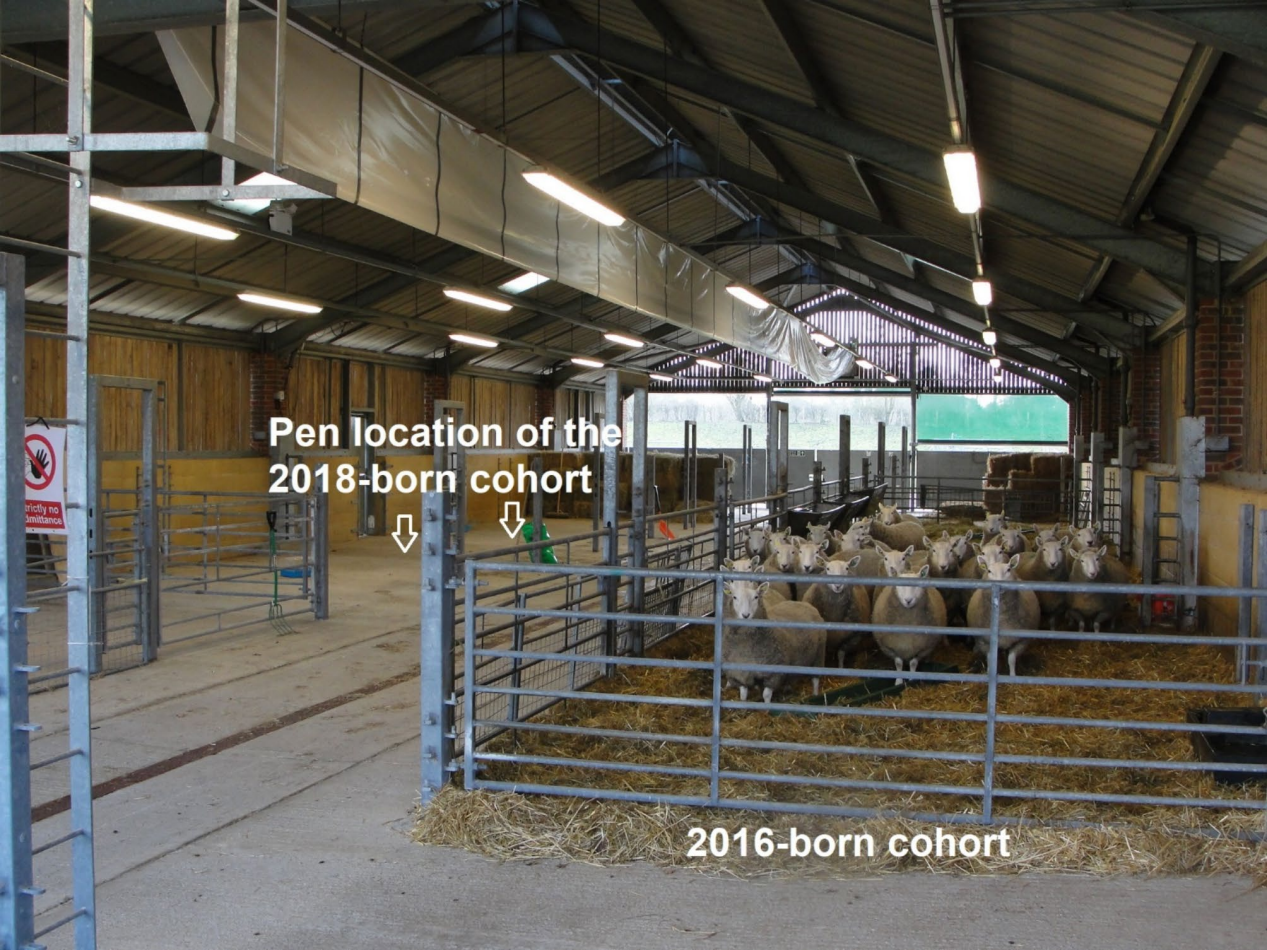
Barn housing the sheep Pen of the 2016-born cohort sheep, 659 days after the first sheep moved in the barn and 74 days prior to the arrival of the first 2018-born cohort sheep that were penned at the left of the barn, indicated by the arrows

Animal accommodation and determination of scrapie status was as described in the previous study [4]. The study was in the same rectangle-shaped barn with natural air circulation, with concrete walls up to 1.5 m height and then hit and miss wooden boards on two opposite sides and two entrances/ exits on the other two opposite sides with metal gates. Metal fencing, posts and hurdles were used to create a pen for sheep within the barn, with straw as deep litter and hay in racks fed ad libitum. Staff entering the barn wore disposable overalls with boots dedicated to the farm, and foot dips with sodium hypochlorite (20,000 ppm free chlorine) were at the entrance, which were changed no later than every 5 days. Similar to the previous study, the barn was decontaminated by four times 1 h application of sodium hypochlorite solution, except for metalwork which was soaked in baths for 4 h in the same solution. Exposure to sodium hypochlorite included the wooden hit and miss paneling, which was not done in the previous experiment, but not the ceiling because of the difficulty spraying disinfectant safely at this height. The last remaining five sheep from the previous experiment that were scrapie-positive had left the barn 18 days earlier, whilst the last sheep affected by scrapie grazing in the adjacent pasture were removed 440 days prior to the last five sheep leaving the barn (458 days before the new sheep moved in). The study was carried out in two phases because of the limited, seasonal availability of Cheviot sheep with a prion protein genotype associated with high susceptibility to scrapie (V_136_R_154_Q_171_ homozygous). These sheep were derived from APHA’s own flock, which was founded with sheep originally imported from New Zealand, which is free from classical scrapie [5], and culled sheep are regularly tested for scrapie to confirm its scrapie-free status. Two birth cohorts were used: 25 sheep in the 2016-born cohort (see Fig. 1) and 21 sheep in the 2018-born cohort.

The 2018-born cohort was introduced whilst sheep from the 2016-born cohort were still in the barn. The cohorts were kept in different sites of the pen and never mixed. The area, which accommodated the 2016-born cohort was hosed down with water after removal of all bedding, decontaminated with sodium hypochlorite as per protocol and then again washed down with water, carefully avoiding creation of any mist that may spread to the other area of the barn accommodating the 2018-born cohort. All sheep were introduced to the barn with their dams as lambs aged between 2 and 20 days. Dams were removed at weaning age of approximately 90 days and tested for scrapie by immunohistochemistry (IHC) on brain and peripheral tissues. Rat monoclonal antibody R145 was used for IHC using an established protocol [6].

Rectal biopsies were taken from sheep and recto-anal mucosa-associated lymphoid tissue (RAMALT) examined by IHC to determine scrapie status [7]. After local anaesthesia of the rectum (EMLA, AstraZeneca), a sample of the rectal mucosa was taken from each lamb starting at 180 dpe. This was repeated every 6 months in the 2016-born cohort and after a year in the 2018-born cohort because the results (see below) implied that infection would not be detectable early in the incubation period; it was done six-monthly thereafter. Sampling was repeated if the number of follicles in the examined sections (including serial sections from the wax block) were less than 1, which was considered inadequate to reach a diagnosis.

Any scrapie-positive sheep was removed and culled, except for four sheep of the 2018-born cohort, which were removed from the barn and retained for another study until the development of clinical disease. Those were culled at clinical esnd-stage (progressive signs of pruritus, ataxia and/ tremor) based on a short clinical assessment protocol [8]. Sheep with a negative RAMALT result were culled not earlier than 868 dpe, when it was considered unlikely that they were infected. After euthanasia with anaesthetic overdose (Somulose, Dechra; 1 ml/ kg body weight administered intravenously), brain (obex) and peripheral lymphoid tissues (medial retropharyngeal and mesenteric lymph nodes, palatine tonsil, distal ileum, RAMALT) were collected for IHC. A subset (left side of the brain parts and lymph nodes of the left site) was frozen at -80 °C.

Statistical analysis was limited to descriptive statistics, such as mean or median if data were not normally distributed. The two-tailed Fisher exact test was used to compare the number of scrapie-infected sheep in the 2016 and 2018-born cohorts with *P* < 0.05 considered significant. All calculations were done with Statistica (TIBCO, version 14.0.0.15).

## Results

**Fig. 2:**
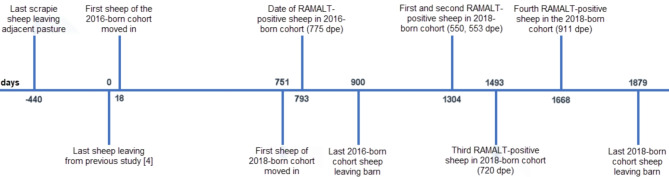
Timeline of events

Detailed results of the sheep are provided in additional file 1 - Sheep data. Figure 2 provides a timeline of the events.

All ewes, which were kept in the barn until weaning age of their lambs, were scrapie-negative. Median barn exposure time was 65 days for the dams of the 2016-born cohort and 107 days for the dams of the 2018-born cohort.

### 2016-born cohort

**Fig. 3:**
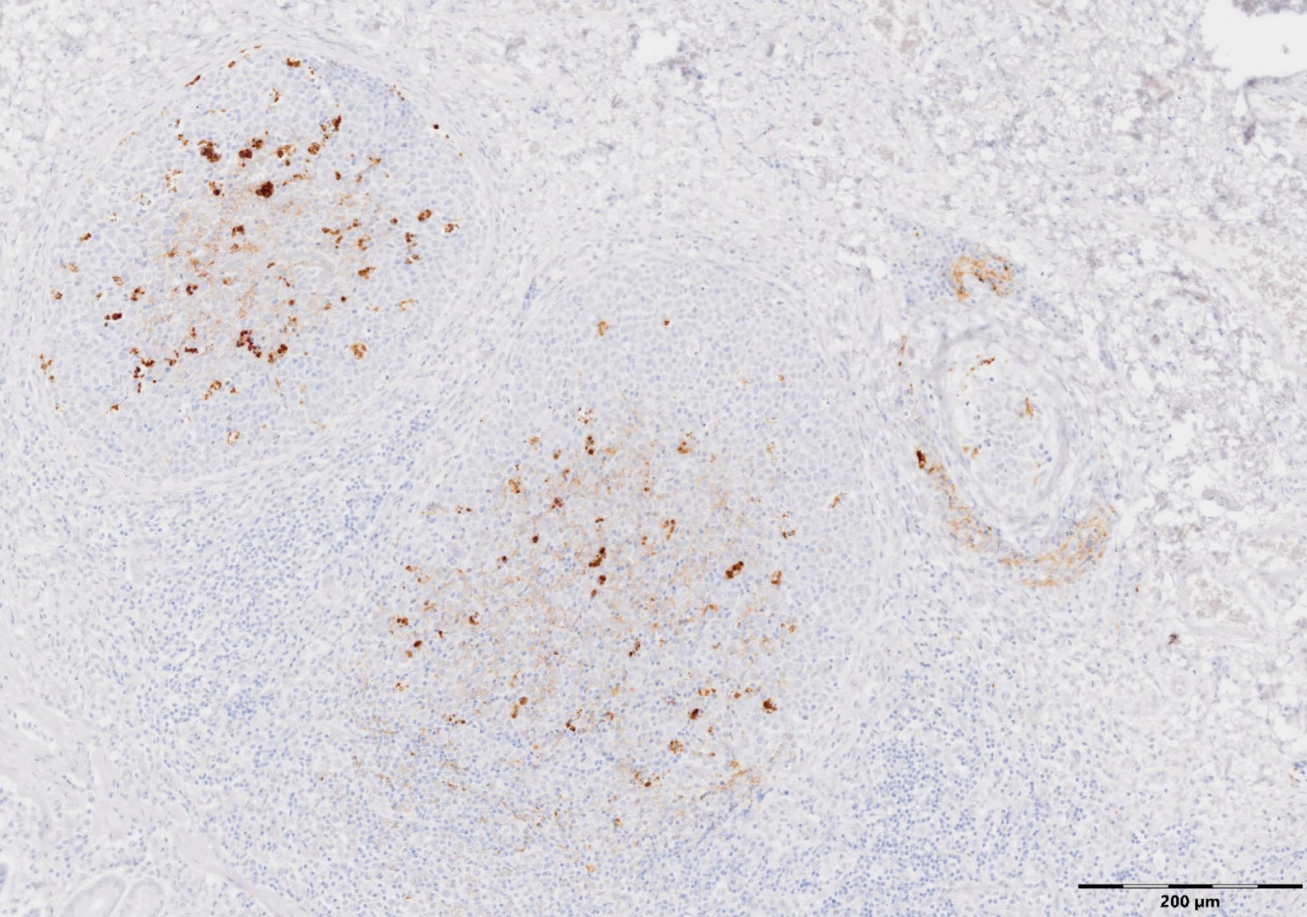
PrP^Sc^ detection in RAMALT

In the 2016-born cohort, only one of 25 sheep (4%) had detectable scrapie-associated prion protein (PrP^Sc^) in the rectal biopsy (see Fig. 3).

Rectal biopsy of 2016-born cohort sheep 6004 at 775 dpe. PrP^Sc^ immunolabelling is visible in lymphoid follicles, located in tingible body macrophages and follicular dendritic cells. Antibody R145.

This was at 775 dpe, by which time sheep from the 2018 cohort had already been in the other side of the barn for 42 days. Examined tissues by IHC from this sheep after cull confirmed PrP^Sc^ was restricted to lymphoid tissue. Remaining sheep were culled from 868 dpe, with no detectable PrP^Sc^ in RAMALT whilst alive, and were all scrapie-negative.

### 2018-born cohort

In the 2018-born cohort, one lamb died of an intercurrent disease at 32 dpe and was scrapie-negative. Subsequently, four of the 20 remaining sheep had detectable PrP^Sc^ in RAMALT at 550, 553, 720 and 911 dpe respectively. These were retained for a separate study and were culled with clinical disease at 908, 2,084, 1,328 and 1,495 dpe respectively. Scrapie was confirmed in brain and lymphoid tissue. The remaining 16 sheep with no PrP^Sc^ in RAMALT were culled from 1,105 dpe. None had scrapie confirmed.

The incidence of infection in the 2016 and 2018-born cohorts was not significantly different (*P* = 0.16, Fisher’s exact test), although it was significantly lower compared to the previous study [4] when 24 of 25 sheep became infected (*P* < 0.001).

## Discussion

Cleaning and disinfection (C&D) are imperative in any animal disease outbreak to reduce contamination and prevent re-infection of animals newly introduced animals. Unfortunately, the infectious agent responsible for TSEs, including scrapie, is extremely resistant to disinfection and other inactivation protocols and can persist in the environment for many years, which makes this very challenging [1]. Validated disinfection protocols based on experimental settings commonly applied use either sodium hydroxide or sodium hypochlorite, which are extremely corrosive, hazardous to humans and animals, and its use in a farm setting is questionable. Indeed, previous studies utilising the same farm as the one used in the current study have shown that effective decontamination of an animal barn that housed scrapie-affected sheep is virtually impossible because infection still re-occurs [2, 4]. It was hypothesized that the most likely cause of re-infection was dust from contaminated surroundings because of the detection of prions in dust samples collected in the barn [4]. However, a study assessing infectivity of field furniture suggested that there may be gradual reduction of prion activity through the weathering process (repeated cycle of environmental heat and cold) [9]. If this was the case, it should be possible to achieve sufficient decontamination over time if the disinfection protocol in the barn was continued. The current study aimed to assess whether inactivation may be possible over time by repeated decontamination using longer exposure times than recommended (4 × 1 h rather than a single hour [1]).

It was acknowledged that it may be difficult to assess contamination at very low levels by bioassay because sample size needs to increase. Twenty-five sheep used in the 2016-born cohort would have been sufficient to detect an infection rate of about 11% with 95% confidence. It was unfortunate that infection of a single sheep was only detected at 775 dpe, by which time we had already moved a second group into the barn. Retrospectively, it might have been better to wait until all sheep in the 2016-born had been culled and examined to determine scrapie status and then decide on the future, e.g. another cycle of C&D with introduction of sheep or discontinue, but it would not have taken into account that results may differ between groups at different time points when contamination may increase (due to more dust) or decrease (due to more inactivation of prions from the outside because of the weathering process). There appeared to be an increase in infection rate although the difference between the 2016- and 2018-born cohort was not significant. It is possible that this was a result of gradual increase in contamination, caused by the single scrapie-positive sheep at 775 dpe in the 2016-born cohort, by the scrapie-positive sheep in the 2018-cohort and resulting contamination of the whole barn, by dust from the outside, by dust from the ceiling that was not disinfected or by a combination of these. The first positive animal in the 2018-born cohort was detected by rectal biopsy examination at 550 dpe, which was 358 days after the previous, negative biopsy and considerably earlier than in the 2016-born cohort (775 dpe), which may imply a higher level of prion contamination of the barn. It has been shown that in natural infection prions can be detected by immunohistochemistry in lymphoid tissue in VRQ/VRQ lambs from 2 months of age [10, 11] and may be detected in the enteric nervous system of the small intestine up to 9 months before being detected in rectal tissue [12]. As shedding of the infectious agent from infected sheep may occur very early after infection, particularly in VRQ/VRQ sheep with an extensive lymphoid prion spread, it is likely that infected animals contributed to the subsequent infection of other sheep in the pen or spread of prions in dust prior to their removal from the barn. However, it would not explain the infection of the single sheep in the 2016-born cohort, which was by prions from the outside or by prions within the barn that were not inactivated during the disinfection process. Nevertheless, there was a significant reduction in infection incidence compared to the previous study with the same decontamination regime, which caused infection of 24 or 25 sheep [4], even though complete inactivation of prions was not achieved.

The comparatively long period between exposure and first detection of PrP^Sc^ in RAMALT (775 days) and low infection rate (1 of 25) in the 2016-born cohort is suggestive of low infectious titre contamination with the scrapie agent. In 2002, six of eight lambs exposed to pasture on this farm from 2 days of age for 12 months without contact to scrapie-affected sheep had a median survival time of 794 days [13], i.e. they were at or close to clinical end-stage by the time the sheep in the 2016 cohort was just confirmed to be infected. The data from the 2018-born cohort suggest that it may take a minimum of 358 days from the day of first detection of PrP^Sc^ in RAMALT to clinical end-stage. Exposure of VRQ heterozygous sheep or sheep without a VRQ allele may result in an even longer incubation period, and infection may go unnoticed for some time leading to the erroneous assumption that sheep are free from the disease. As scrapie surveillance is generally restricted to examination of brain only, sheep at an earlier stage of infection when PrP^Sc^ spread to the central nervous system has not yet occurred may be missed.

In conclusion, this study has shown that repeated disinfection with sodium hypochlorite, even using extended decontamination times, did not prevent re-infection so that there is a risk of re-infection if sheep with susceptible genotypes are re-introduced. It is not known whether decontamination was ineffective or recontamination occurred from various sources.

### Limitations

- Source of contamination could not be established, which would potentially help to suggest prevention strategies.
- Study design was not ideal due to the unpredictable nature of prion diseases (long incubation period until detection of infection in the first cohort but unexpectedly shorter in the second cohort).

## Electronic supplementary material

Below is the link to the electronic supplementary material.

Supplementary Material 1: Sheep data.xlsx. This file lists all sheep-related data, such as breed, sex, age at exposure and scrapie status based on rectal biopsy and brain testing.

## Data Availability

Data are provided within the manuscript or supplementary information files.

## Abbreviations

APHA: Animal and Plant Health Agency
C&D: Cleaning and disinfection
dpe: Days post exposure
IHC: Immunohistochemistry
PrP^Sc^: Scrapie-associated prion protein
RAMALT: Recto-anal mucosa-associated lymphoid tissue
sPMCA: Serial protein misfolding cyclic amplification
